# Demonstração da relação entre características sociodemográficas e depressão em pacientes com síndrome do túnel do carpo

**DOI:** 10.1055/s-0045-1809527

**Published:** 2025-06-14

**Authors:** Şule Göktürk, Yasin Göktürk, Belgin Oral, Eda Başmısırlı

**Affiliations:** 1 Departamento de Neurocirurgia, Kayseri City Hospital, University of Health Sciences, Kayseri, República da Turquia.; 2 Departamento de Saúde Pública, Clínica de Doenças Ocupacionais, Kayseri City Hospital, University of Health Sciences, Kayseri, República da Turquia.; 3 Departamento de Nutrição e Dietética, Faculty of Health Sciences, Nuh Naci Yazgan University, Kayseri, República da Turquia.

**Keywords:** ansiedade, depressão, síndrome do túnel do carpo, anxiety, carpal tunnel syndrome, depression

## Abstract

**Objetivo:**

Apresentar as características clínicas, epidemiológicas e socioeconômicas de indivíduos com sintomas de síndrome do túnel do carpo (STC) e depressão.

**Métodos:**

O Inventário de Depressão de Beck (IDB) foi administrado a 100 pacientes diagnosticados com STC e sua relação entre ansiedade e depressão foi investigada juntamente com as características sociodemográficas dos pacientes.

**Resultados:**

A depressão foi identificada em 30,9% dos 68 pacientes, em sua maioria mulheres. As pontuações no IDB foram significativamente maiores em pacientes com condição socioeconômica baixa, má nutrição e tabagismo ( *p* < 0,05). Além disso, a frequência de sintomas depressivos foi significativamente maior em pacientes com dor lombar e cervical crônica além da STC, com uso regular de analgésicos e que já haviam sido submetidos a alguma cirurgia ( *p* < 0,05).

**Conclusão:**

Depressão e ansiedade são prevalentes entre indivíduos diagnosticados com STC. O tratamento eficaz, incluindo o controle da depressão e ansiedade comórbidas, é crucial para bons desfechos.

## Abstract (en)

**Objective:**

To present the clinical, epidemiological and socioeconomic characteristics of individuals with symptoms of carpal tunnel syndrome (CTS) and depression.

**Methods:**

The Beck Depression Inventory (BDI) was administered to 100 patients diagnosed with CTS, and its relationship between anxiety and depression was investigated alongside the sociodemographic characteristics of the patients.

**Results:**

Depression was identified in 30.9% of the 68 patients, the majority of whom were women. The BDI scores were found to be significantly higher in patients with low socioeconomic status, poor nutrition, and smoking ( *p* < 0.05). Furthermore, the frequency of depressive symptoms was found to be significantly higher in patients who had chronic low back and neck pain along with CTS, with regular use of analgesics, and who had previously undergone any surgery ( *p* < 0.05).

**Conclusion:**

Depression and anxiety are prevalent among individuals diagnosed with CTS. Effective treatment, including the management of comorbid depression and anxiety, is crucial for optimal patient outcomes.

## Introdução

A síndrome do túnel do carpo (STC) é uma decorrente da compressão do nervo mediano em vários níveis dentro do túnel do carpo, no punho. É a neuropatia periférica mais prevalente nos membros superiores. 1 Apesar das incertezas sobre sua patogênese, a STC é observada com maior frequência em indivíduos de meia-idade e do gênero feminino. Também é possível que a STC ocupacional ocorra em indivíduos submetidos à pressão no retináculo flexor, localizado na parte interna da mão. No entanto, é importante ressaltar que a prevalência da STC também é alta em indivíduos com obesidade, gravidez, diabetes, doenças reumatológicas e em uso crônico de álcool.

A STC ocupacional é um fenômeno raro e é essencial excluir todas as outras possíveis causas, em especial fatores como obesidade, antes de atribuir a doença a fatores laborais. Os mecanismos mais prevalentes na patogênese da STC são compressão mecânica, insuficiência microvascular e teorias de vibração. A teoria da compressão mecânica postula que os sintomas da STC são causados pela compressão do nervo mediano dentro do túnel do carpo. 1

A prevalência da STC e sua distribuição na sociedade podem variar de maneira considerável, com estimativas entre 0,125 e 1% e entre 5 e 16%. A etiologia ou causa subjacente também é um fator significativo na determinação de sua incidência. 2 As duas formas principais de STC são aguda e crônica. A primeira é menos comum e decorre do aumento súbito na pressão no interior do túnel do carpo. A doença foi descrita pela primeira vez por Sir James Paget em 1854 no contexto de uma fratura do rádio. 3 Silverstein et al. investigaram a correlação entre movimentos repetitivos/de alta força e STC em uma coorte de 652 trabalhadores em 39 profissões, de 7 setores distintos. Seus achados indicaram que movimentos repetitivos podem representar um fator de risco significativo para a STC. No entanto, os autores também sugeriram que alta força e repetição por si só podem não ser suficientes para induzir a STC. 4

Os sintomas clássicos da STC incluem parestesia, dormência e formigamento nas áreas inervadas pelo nervo mediano, principalmente à noite. O diagnóstico da STC pode empregar vários testes, como os sinais de Tinel, de Phalen, do punho quadrado, do punho fechado e do movimento rápido, assim como o diagrama da mão de Katz, o teste de flexão e extensão do punho, o teste de provocação por pressão e o teste do torniquete.

Os sintomas podem variar dependendo da gravidade da doença. Na fase inicial, os sintomas são decorrentes do comprometimento do componente sensorial do nervo mediano. Por outro lado, na fase subsequente, os sintomas são uma consequência do acometimento das fibras motoras. O sintoma mais observado é a dor com sensação de queimação, acompanhada de parestesia e dormência na distribuição do nervo mediano no punho distal. Os dedos afetados são o polegar, o indicador, o médio e a metade radial do anular. Os pacientes geralmente relatam dor noturna e parestesia, com tentativas de aliviar os sintomas apertando as mãos ou mudando de posição. O diagnóstico pode ser estabelecido por meio de eletromiografia (EMG), ultrassonografia (USG) e ressonância magnética (RM). 5

Há diversas opções de tratamento cirúrgico e não cirúrgico para a STC. Os métodos não cirúrgicos são eficazes em pacientes com STC branda a moderada. A injeção de corticosteroides no punho é um método frequentemente empregado por fisioterapeutas ou médicos especialistas em dor. Embora os sintomas possam piorar temporariamente, o tratamento pode proporcionar alívio completo ou significativo da dor em 60 a 70% dos pacientes em semanas ou anos. 6 7 8 Em procedimentos cirúrgicos, o ligamento transverso do carpo é incisado para relaxamento do nervo, em uma operação de descompressão.

Os indivíduos diagnosticados com STC podem perceber sua doença como uma barreira à continuidade do emprego, o que pode levar a desafios financeiros. Além disso, podem sentir desconforto físico e sensação de diminuição de força, o que pode contribuir para uma série de dificuldades emocionais e psicológicas. Essas podem incluir sintomas depressivos, como ansiedade, redução do interesse e do envolvimento nas atividades diárias, assim como sentimentos de insatisfação e tristeza. Foi documentado que indivíduos com doenças físicas são mais propensos a ter ansiedade e depressão. 9 Também foi demonstrado que doenças de saúde mental, como depressão e ansiedade, podem gerar limitações funcionais em pacientes com distúrbios musculoesqueléticos dos membros superiores. 10

O objetivo deste estudo foi examinar a relação entre o estado de ansiedade e depressão e a doença em pacientes com STC usando o inventário de depressão de Beck (IDB).

## Materiais e Métodos

Este estudo foi realizado na Clínica de Neurocirurgia do hospital entre março e junho de 2024. Aqueles com STC atendidos na clínica ambulatorial foram primeiramente informados sobre o estudo e assinaram o termo de consentimento livre e esclarecido, de acordo com os princípios da Declaração de Helsinque. O Comitê de Ética de nosso hospital aprovou o estudo sob número 76397871 em 15 de março de 2024.

### Delineamento Experimental

A amostra do estudo foi calculada como 100 a partir de informações da literatura, dos programas estatísticos Number Cruncher Statistical System (NCSS) 2007 e o Power Analysis and Sample Size (PASS) 2008 (NCSS LLC., Kaysville, UT, EUA). A população do estudo incluiu 100 pacientes com STC, com idades entre 18 e 72 anos, incluindo aqueles com doença branda, moderada e grave, independentemente do gênero. Os pacientes responderam a um questionário com 21 perguntas sobre seus dados sociodemográficos, incluindo idade, gênero, local de residência, educação e situação econômica. Quatro das perguntas eram abertas e focavam no comportamento em relação ao tabagismo e ao uso de álcool, exercícios e nutrição. Além disso, incluiu-se o Inventário de Depressão de Beck (IDB), com 21 perguntas. Os dados foram coletados por um único pesquisador em entrevista presencial. A altura do paciente (em centímetros), o peso corporal (em quilogramas) e o índice de massa corporal (IMC) foram calculados em uma balança de pesagem e medição com precisão de 0,01 kg, com o paciente descalço e vestindo roupas esportivas.

### Critérios de Exclusão

A população do estudo foi composta por indivíduos com doenças neurológicas com acometimento do sistema nervoso central e resultando em déficits neurológicos nos membros superiores. Além disso, a coorte inclui indivíduos diagnosticados com polineuropatia.

### Inventário de Depressão de Beck

O IDB é frequentemente utilizado em nosso país. Composto por 21 questões, é um instrumento de pesquisa com quatro opções de resposta que gera pontuações entre 0 e 3. A validade e a confiabilidade do IDB em turco foram estabelecidas por Hisli. 11

### Análise Estatística

Os dados foram registrados no programa IBM SPSS Statistics for Windows (IBM Corp., Armonk, NY, EUA), versão 22.0, e as análises foram realizadas no mesmo programa. As estatísticas descritivas incluíram frequência, porcentagem, média, desvio padrão (DP), mediana e valores mínimo e máximo (min.–max.). A análise estatística dos dados categóricos aplicou a correção de Yates para valores abaixo de 25 e o teste exato de Fisher foi empregado para valores abaixo de cinco. O teste de Kolmogorov-Smirnov verificou se os dados apresentavam distribuição normal. Em grupos binários, nos quais os dados quantitativos não estavam em distribuição normal em grupos independentes, o teste U de Mann-Whitney foi utilizado. Nos grupos com mais de duas categorias, o teste de Kruskall-Wallis (teste *post hoc* de Dunn) foi aplicado. A diferença estatística significativa foi definida por *p* < 0,05.

## Resultados

A idade média dos 100 pacientes com STC que participaram do estudo foi de 44,76 ± 13,45 (mín.–máx.: 18–72) anos e 68% da coorte era composta por mulheres. A pontuação média do IDB dos pacientes foi 11,99 ± 7,42 (mín.–máx.: 0–34). Quando o ponto de corte foi considerado igual ou maior a 17, a frequência de sintomas depressivos foi de 26%. Embora as pontuações do IDB tenham sido relativamente baixas entre os universitários, foram significativamente maiores entre aqueles que definiram a sua situação econômica como pobre, residiam em áreas rurais e desempregados ( *p* < 0,05), como mostrado na Tabela 1 . Em nosso estudo, a frequência de sintomas depressivos foi significativamente maior em pacientes com STC que descreveram sua dieta como irregular, não praticavam atividade física regular, haviam recebido ajuda psicológica prévia e com uso prévio de antidepressivos ( *p* < 0,05). Além disso, as pontuações do IDB foram significativamente menores em fumantes e usuários de álcool ( *p* < 0,05). Entretanto, a diferença observada não foi estatisticamente significativa ( *p* > 0,05), como pode ser visto na Tabela 2 .

**Tabela 1:** Achados sociodemográficos e pontuações do IDB dos pacientes com STC

Variáveis		n	Pontuação no IDB			Valor de *p*	Frequência de depressão		Valor de *p*
			Mediana	Mín.–máx.	Média ± DP		Positiva: n (%)	Negativa: n (%)	
**Gênero**	Feminino	68	10,5	0–37	12,0 ± 7,3	0,827*	21 (30,9)	47 (69,1)	0,168***
	Masculino	32	11,0	0–34	11,9 ± 7,3		5 (15,6)	27 (84,4)	
**Idade (anos)**	≤ 40	42	11,0	0–26	11,4 ± 6,5	0,622*	8 (19,0)	34 (81,0)	0,264***
	> 40	58	10,0	0–34	12,4 ± 8,0		18 (31,0)	40 (69,0)	
**Escolaridade**	Abaixo do ensino médio	66	12,5	0–34	12,8 ± 7,8	0,029**	20 (30,3)	46 (69,7)	0,144****
	Ensino médio	25	11,0	3–26	11,8 ± 6,5		6 (24,0)	19 (76,0)	
	Ensino superior	9	6,0	0–13	6,1 ± 4,0		0 (0,0)	9 (100,0)	
**Condição econômica**	Boa	35	8,0	0–27	10,5 ± 7,2	0,032**	6 (17,1)	29 (82,9)	0,037****
	Média	61	11,0	0–29	12,2 ± 7,1		17 (27,9)	44 (72,1)	
	Ruim	4	20,5	13–34	22,0 ± 8,7		3 (75,0)	1 (25,0)	
**Tipo de família**	Pequena	69	10,0	0–34	11,8 ± 7,8	0,488*	17 (24,6)	52 (75,4)	0,828***
	Extensa	31	13,0	0–26	12,5 ± 6,7		9 (29,0)	22 (71,0)	
**Habitação**	Cidade	68	9,0	0–34	10,9 ± 7,3	0,013*	13 (19,1)	55 (80,9)	0,041***
	Município	32	14,5	0–27	14,4 ± 7,2		13 (40,6)	19 (59,4)	
**Empregado**	Sim	39	8,0	0–26	9,2 ± 6,3	0,001*	4 (10,3)	35 (89,7)	0,008***
	Não	61	13,0	0–34	13,8 ± 7,6		22 (36,1)	39 (63,9)	

**Abreviações** : DP, desvio padrão; IDB, Inventário de Depressão de Beck; Mín.–máx., valores mínimos e máximos; STC, síndrome do túnel do carpo.

**Notas:** *Teste U de Mann-Whitney. **Teste de Kruskal-Wallis. ***Teste do Qui quadrado de correção de Yates. ****Teste exato de Fisher.

**Tabela 2:** Condições de saúde e comportamental e pontuação do IDB em pacientes com STC

Variáveis		n	Pontuação no IDB			Valor de *p*	Frequência de depressão		Valor de *p*
			Mediana	Mín.–máx.	Média ± DP		Positiva: n (%)	Negativa: n (%)	
**Doença crônica**	Sim	44	11,5	0–34	12,9 ± 8,5	0,470*	15 (34,1)	29 (65,9)	0,160***
	Não	56	10,0	0–26	11,3 ± 6,4		11 (19,6)	45 (80,4)	
**Medicação regular**	Em uso	42	12,0	0–29	12,9 ± 7,9	0,314*	15 (35,7)	27 (64,3)	0,098***
	Não	58	10,0	0–34	11,3 ± 7,0		11 (19,0)	47 (81,0)	
**Doença crônica em familiares**	Sim	28	11,0	0–29	11,3 ± 7,4	0,612*	6 (21,4)	22 (78,6)	0,692***
	Não	72	10,5	0–34	12,3 ± 7,5		20 (27,8)	52 (72,2)	
**Estado nutricional**	Regular	74	10,0	0–27	11,1 ± 6,7	0,105*	15 (20,3)	59 (79,7)	0,028***
	Irregular	26	13,0	0–34	14,4 ± 8,8		11 (42,3)	15 (57,7)	
**Exercícios regulares**	Sim	22	9,0	3–24	9,7 ± 5,5	0,122*	2 (9,1)	20 (90,9)	0,041***
	Não	78	12,0	0–34	12,6 ± 7,8		24 (30,8)	54 (69,2)	
**Tabagismo**	Sim	38	8,5	0–34	10,4 ± 7,8	0,049*	7 (18,4)	31 (81,6)	0,264***
	Não	62	13,0	0–29	12,9 ± 7,1		19 (30,6)	43 (69,4)	
**Alcoolismo**	Sim	12	8,5	0–34	9,6 ± 6,2	0,203*	1 (8,3)	11 (91,7)	0,177****
	Não	88	12,0	3–26	12,3 ± 7,5		25 (28,4)	63 (71,6)	
**Ajuda psicológica prévia**	Sim	9	18,0	5–27	16,4 ± 8,1	0,082*	5 (55,6)	4 (44,4)	0,049****
	Não	91	10,0	0–34	11,5 ± 7,3		21 (23,1)	70 (76,9)	
**Uso prévio de antidepressivos**	Sim	9	23,0	8–27	19,3 ± 7,0	0,003*	6 (66,7)	3 (33,3)	0,009****
	Não	91	10,0	0–34	11,3 ± 7,1		20 (22,0)	71 (78,0)	
** IMC (kg/m ^2^ ) **	< 25	16	12,0	0–34	12,9 ± 9,4	0,148**	3 (18,8)	13 (81,3)	0,123
	25–29,9	44	9,0	0–24	10,3 ± 6,6		8 (18,2)	36 (81,8)	
	≥ 30	40	13,0	0–29	13,4 ± 7,3		15 (37,5)	25 (62,5)	

**Abreviações** : DP, desvio padrão; IDB, inventário de depressão de Beck; IMC, índice de massa corporal; Mín.–máx., valores mínimos e máximos; STC, síndrome do túnel do carpo.

**Notas:** *Teste U de Mann-Whitney. **Teste de Kruskal-Wallis. ***Teste do Qui quadrado de correção de Yates. ****Teste exato de Fisher.

A prevalência de sintomas depressivos foi significativamente maior em pacientes STC que apresentavam dor lombar e cervical crônica, usavam analgésicos regulares devido a essa dor e que já haviam sido submetidos a alguma cirurgia ( *p* < 0,05), como mostrado na Tabela 3 .

**Tabela 3:** Dor lombar/cervical crônica e pontuação do IDB em pacientes com STC

Variáveis		*n*	Pontuação no IDB			Valor de *p*	Frequência de depressão		Valor de *p*
			Mediana	Mín.–máx.	Média ± DP		Positiva: n (%)	Negativa: n (%)	
**Dor cervical crônica**	Sim	47	13,0	0–34	14,2 ± 8,2	0,009*	21 (44,7)	26 (55,3)	<0,001**
	Não	53	9,0	0–29	10,0 ± 6,1		5 (9,4)	48 (90,6)	
**Dor lombar crônica**	Sim	41	11,0	0–34	13,4 ± 8,7	0,232*	17 (41,5)	24 (58,5)	0,007**
	Não	59	11,0	0–29	11,0 ± 6,3		9 (15,3)	50 (84,7)	
**Uso de analgésicos para dor lombar/cervical crônica**	Sim	50	12,5	0–27	13,1 ± 7,2	0,102*	18 (36,0)	32 (64,0)	0,040**
	Não	50	9,5	0–34	10,8 ± 7,5		8 (16,0)	42 (84,0)	
**Cirurgia anterior**	Sim	49	13,0	0–34	13,8 ± 8,2	0,029*	18 (36,7)	31 (63,3)	0,030**
	Não	51	9,0	0–24	10,2 ± 6,2		8 (15,7)	43 (84,3)	

**Abreviações** : DP, desvio padrão; IDB, inventário de depressão de Beck; Mín.–máx., valores mínimos e máximos; STC, síndrome do túnel do carpo.

**Notas** : *Teste U de Mann-Whitney; **Teste exato de Fisher.

## Discussão

O presente estudo revelou que a prevalência de sintomas depressivos foi de 26%. Notavelmente, observou-se que as pontuações do IDB eram relativamente baixas entre os universitários. No entanto, descobriu-se que eram significativamente maiores entre indivíduos que se autoidentificaram como economicamente desfavorecidos, residentes de áreas rurais e desempregados.

Em pacientes com STC, dor, parestesia e sensações de formigamento na área inervada prejudicam a qualidade de vida. Essa condição tem um impacto profundo na função física em indivíduos com hipoestesia persistente e comprometimento motor. Também pode dar origem a problemas de saúde mental, como ansiedade ou depressão. 12 McCallum et al. mostraram uma associação dos níveis de ansiedade e depressão com a presença de dor. 13 Este achado é corroborado por pesquisas anteriores que examinaram a incidência de doenças mentais em indivíduos com dor crônica. 14 Além disso, foi demonstrado que circunstâncias sociais e atividades físicas que influenciam a saúde podem estar relacionadas à saúde mental em vários distúrbios musculoesqueléticos e neurológicos. 15 16

De acordo com Adjibade et al., 17 pacientes que exibiram múltiplos comportamentos de estilo de vida saudável (ou seja, não fumar, baixo consumo de álcool, atividade física regular, dieta saudável e IMC normal) demonstraram menor probabilidade de apresentar sintomas depressivos em comparação aos com dois ou menos comportamentos de estilo de vida saudável. Em nosso estudo, a frequência de sintomas depressivos foi significativamente maior em indivíduos que descreveram sua dieta como irregular, não praticavam atividade física regular, já haviam recebido ajuda psicológica e já haviam usado antidepressivos. Ademais, as pontuações do IDB foram significativamente menores entre fumantes.

Em um estudo de 2020, Paiva Filho et al. 18 avaliaram a prevalência de ansiedade e depressão em pacientes com STC. Embora uma associação entre IMC e maior índice de depressão ou ansiedade não tenha sido observada quando considerados isoladamente, quase ⅔ dos pacientes apresentaram valores acima da normalidade. Em seu estudo, as características estatisticamente associadas aos sintomas de ansiedade e depressão em pacientes com STC, independentemente de outras características, foram gênero, tabagismo e renda familiar. Em nosso estudo, valores menores de IDB também estavam relacionados a uso de álcool, mas a diferença não foi estatisticamente significativa.

A relação entre sintomas depressivos e desfechos cirúrgicos na STC é de particular interesse, dada a alta prevalência de ambas as doenças, particularmente em mulheres. 19 Neste estudo, a depressão foi identificada em 30,9% das 68 pacientes do gênero feminino. As pontuações do IDB foram relativamente baixas entre universitário, mas significativamente maiores entre aqueles que descreveram sua situação econômica como ruim, residiam fora da cidade (em municípios ou vilarejos) e estavam desempregados. Mais uma vez, a literatura existente indica uma associação estatisticamente significativa entre tabagismo e renda familiar e ansiedade em pacientes com STC. 19 A depressão por si só tinha associação estatisticamente significativa com a renda familiar em pacientes com STC. Estudos mostram que transtornos como depressão e ansiedade podem contribuir para limitações funcionais em pacientes com distúrbios musculoesqueléticos dos membros superiores. 20

Além disso, nosso estudo demonstrou que a prevalência de sintomas depressivos foi significativamente elevada em pacientes com dor lombar e cervical crônica em pacientes com STC, com uso regular de analgésicos, que haviam passado por procedimentos cirúrgicos anteriores ( *p* < 0,05).

É de grande importância compreender as características sociodemográficas de indivíduos com STC e transtornos de humor, particularmente ansiedade e depressão, para fornecer o tratamento ideal e facilitar recuperação após a intervenção cirúrgica. O presente estudo é baseado em um pequeno número de artigos publicados sobre tópicos semelhantes, bem como pela literatura mais ampla sobre o assunto. Seu objetivo foi examinar as características clínicas, epidemiológicas e socioeconômicas de indivíduos que apresentam sintomas de STC, ansiedade e depressão.

## Conclusão

Pessoas com STC apresentaram alta prevalência de sintomas de depressão e ansiedade. O tratamento dessa condição é de extrema importância para a recuperação dos pacientes. Mais pesquisas são necessárias para demonstrar a eficácia de abordagens focadas nas causas orgânicas e patológicas, bem como estressores psicossociais, ansiedade e depressão, no alívio dos sintomas da STC.
